# Specifications of Standards in Systems and Synthetic Biology: Status and Developments in 2019

**DOI:** 10.1515/jib-2019-0035

**Published:** 2019-07-13

**Authors:** Falk Schreiber, Björn Sommer, Gary D. Bader, Padraig Gleeson, Martin Golebiewski, Michael Hucka, Sarah M. Keating, Matthias König, Chris Myers, David Nickerson, Dagmar Waltemath

**Affiliations:** Department of Computer and Information Science, University of Konstanz, Konstanz, Germany; Faculty of IT, Monash University, Clayton, Australia; The Donnelly Centre, University of Toronto, Toronto, Canada; Department of Neuroscience, Physiology and Pharmacology, University College London, London, UK; Heidelberg Institute for Theoretical Studies (HITS), Heidelberg, Germany; California Institute of Technology, Pasadena, CA, USA; Heidelberg University, Heidelberg, Germany; Humboldt-University Berlin, Berlin, Germany; University of Utah, Salt Late City, UT, USA; Auckland Bioengineering Institute, University of Auckland, Auckland, New Zealand; University Medicine Greifswald, Greifswald, Germany

## Abstract

This special issue of the *Journal of Integrative Bioinformatics* presents an overview of COMBINE standards and their latest specifications. The standards cover representation formats for computational modeling in synthetic and systems biology and include BioPAX, CellML, NeuroML, SBML, SBGN, SBOL and SED-ML. The articles in this issue contain updated specifications of SBGN Process Description Level 1 Version 2, SBML Level 3 Core Version 2 Release 2, SBOL Version 2.3.0, and SBOL Visual Version 2.1.

## 1 Introduction

Standards play an important role in Systems and Synthetic Biology. COMBINE (‘COmputational Modeling in BIology’ NEtwork) [1], [2] is a formal entity that coordinates standards development in these fields of research, fosters and moderates discussions, designs and implements dissemination strategies, and organises two annual community meetings each year. HARMONY (Hackathons on Resources for Modeling in Biology) is a workshop and hackathon for the development of libraries, specifications and tool support. The COMBINE forum brings together experts from associated fields of research, discusses applications and further developments of COMBINE standards and hence offers a platform for communication between standards developers and users.

COMBINE describes itself as *“…a network formed by the communities developing standards and formats to share computational models. Working together, it is expected that the federated projects will develop a set of interoperable standards covering all the aspects of computational modelling. Building on the experience of mature projects, which already have stable specifications, software support, user-base and community governance, COMBINE helps foster or support fledging efforts aimed at filling gaps or new needs”* [3].

**Figure 1:**
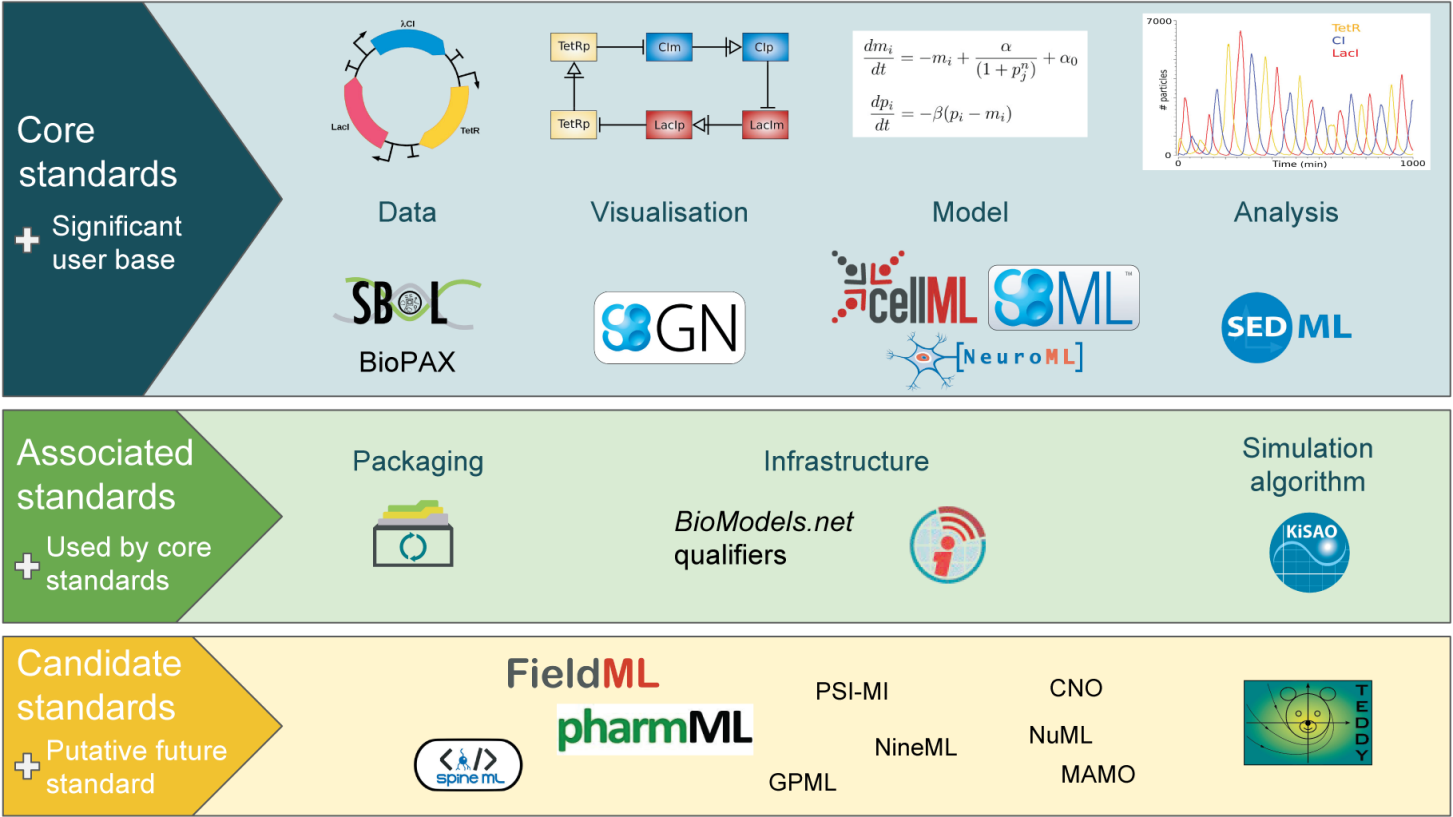
Overview of standards and associated efforts in Systems and Synthetic Biology provided by COMBINE (from [6]).

COMBINE standards and associated initiatives cover a range of topics, see Figure 1. COMBINE is an open initiative and everybody is invited to join. The COMBINE web site https://co.mbine.org/ and COMBINE-related publications [1], [2], [4] provide more information. Please note that this editorial contains similar information to overviews in earlier special issues [5], [6], [7]. We decided to give again a complete overview here as it helps the reader to find all information in one place.

COMBINE published the first collection of Systems and Synthetic Biology standards as a special issue of the Journal of Integrative Bioinformatics in 2015 [5]. Since then a regular special issue of JIB provides updates to COMBINE standards. The latest update was published in 2018 [7]. This special issue presents developments of standards and related initiatives in 2018/19.

## 2 Latest Versions of COMBINE Standards

When using and implementing COMBINE standards, please refer to the following specifications. Note: New specifications are highlighted with *NEW*.

### 2.1 Core Standards

#### 2.1.1 BioPAX (Biological PAthway eXchange)

BioPAX (Biological PAthway eXchange) [8], specification is BioPAX [9].

#### 2.1.2 CellML

CellML [10], specifications are

– CellML 1.1 [11];
– CellML Metadata Framework 2.0 [12].

#### 2.1.3 NeuroML

NeuroML (Neural Open Markup Language) [13], [14], specification is

– NeuroML version 2.0 [13].

#### 2.1.4 SBGN (Systems Biology Graphical Notation)

SBGN (Systems Biology Graphical Notation) [15], specifications are

– *NEW* SBGN Process Description Level 1 Version 2 [16] – which includes new developments such as the addition of equivalence operator, subunit, and annotation glyphs, modification to the usage of submaps, and updates to clarify the use of various glyphs;
– SBGN Entity Relationship Level 1 Version 2.0 [17];
– SBGN Activity Flow Level 1 Version 1.2 [18].

#### 2.1.5 SBML (Systems Biology Markup Language)

SBML (Systems Biology Markup Language) [19], specifications are

– *NEW* SBML Level 3 Core, Version 2 Release 2 [20] – which corrects some errors and clarifies some ambiguities discovered in Release 1. It defines validation rules that determine the validity of an SBML document and provides many examples of models in SBML form;
– SBML Level 3 Core, Version 2 [21];
– SBML Level 3 Package: Flux Balance Constraints, Version 2 [22];
– SBML Level 3 Package: Hierarchical Model Composition, Version 1 [23];
– SBML Level 3 Package: Multistate, Multicomponent and Multicompartment Species, Version 1 Release 1 [24];
– SBML Level 3 Package: Render, Version 1, Release 1 [25];
– SBML Level 3 Package: Qualitative Models, Version 1 [26];
– SBML Level 3 Package: Layout, Version 1 [27];
– SBML Level 3 Package: Groups, Version 1 [28].

#### 2.1.6 SBOL (Synthetic Biology Open Language)

SBOL (Synthetic Biology Open Language) [29], specifications are

– *NEW* SBOL Version 2.3.0 [30] – which includes representing sequence modifications such as insertion, deletion, and replacement, supporting organisation and attachment of experimental data derived from designs and describing numerical parameters of design elements. It includes specifying types of synthetic biology activities, unambiguous locations for sequences with multiple encodings, refinement of a number of validation rules, improved figures and examples, and clarification on issues related to the use of external ontology terms;
– *NEW* SBOL Visual Version 2.1 [31] – which extends the diagram syntax to include methods for showing modular structure and mappings between elements of a system, interactions arrows that can split or join, and new glyphs for indicating genomic context and for stop codons.

#### 2.1.7 SED-ML (Simulation Experiment Description Markup Language)

SED-ML (Simulation Experiment Description Markup Language) [32], specification is

– SED-ML Level 1 Version 3 [33].

### 2.2 Associated Standards

Associated standards provide an additional layer of semantics to COMBINE representation formats. These are:

– COMBINE Archive [34], specification is COMBINE Archive 1.0 [35];
– Identifiers.org URIs [36], community resources to provide persistent identification (no specification);
– Systems Biology Ontology and Kinetic Simulation Algorithm Ontology [37] for controlled vocabularies and semantics in systems biology (no specification);
– BioModels.net qualifiers [38] for representing relation between a model component and the resource used to annotate it (no specification).

## References

[1] Hucka M, Nickerson D P, Bader GD, Bergmann FT, Cooper J, Demir E, et al. Promoting coordinated development of community-based information standards for modeling in biology: the COMBINE initiative. Front Bioeng Biotechnol 2015;3:19. 10.3389/fbioe.2015.00019 PMC4338824 25759811

[2] Myers C, Bader G D, Gleeson P, Golebiewski M, Hucka M, Le Novère N, et al. A brief history of COMBINE. In: Proceedings of the 2017 Winter Simulation Conference. Piscataway, NJ, USA: IEEE Press, 2017:884–95.

[3] Combine. 2017. http://co.mbine.org/. Accessed on 24/06/2019.

[4] Waltemath D, Bergmann FT, Chaouiya C, Czauderna T, Gleeson P, Goble C, et al. Meeting report from the fourth meeting of the computational modeling in biology network (COMBINE). Stand Genomic Sci 2014;9:1285–301. 10.1186/s40793-018-0320-4 PMC6083578 30117501

[5] Schreiber F, Bader GD, Golebiewski M, Gleeson P, Hucka M, Keating SM, et al. Specifications of standards in systems and synthetic biology. J Integr Bioinform 2015;12:258. 10.2390/biecoll-jib-2015-258 PMC5431569 26528556

[6] Schreiber F, Bader GD, Gleeson P, Golebiewski M, Hucka M, Le Novère N, et al. Specifications of standards in systems and synthetic biology: status and developments in 2016. J Integr Bioinform 2016;13:289. 10.2390/biecoll-jib-2016-289 PMC5431574 28187405

[7] Schreiber F, Bader GD, Gleeson P, Golebiewski M, Hucka M, Keating SM, et al. Specifications of standards in systems and synthetic biology: status and developments in 2018. J Integr Bioinform 2018;15:0013. 10.1515/jib-2018-0013 PMC6167034 29596055

[8] Demir E, Cary MP, Paley S, Fukuda K, Lemer C, Vastrik I, et al. The BioPAX community standard for pathway data sharing. Nat Biotechnol 2010;28:935–42. 10.1038/nbt.1666 PMC3001121 20829833

[9] BioPax. 2017. http://www.biopax.org/. Accessed on 24/06/2019.

[10] Cuellar AA, Lloyd CM, Nielsen PF, Bullivant D, Nickerson D, Hunter P. An overview of CellML 1.1, a biological model description language. Simulation 2003;79:740–7.

[11] Cuellar AA, Hedley W, Nelson M, Lloyd CM, Halstead MDB, Bullivant DP, et al. The CellML 1.1 specification. J Integr Bioinform 2015;12:259. 10.2390/biecoll-jib-2015-259 26528557

[12] Cooling MT, Hunter PJ. The CellML metadata framework 2.0 specification. J Integr Bioinform 2015;12:260. 10.2390/biecoll-jib-2015-260 26528558

[13] Cannon RC, Gleeson P, Crook S, Ganapathy G, Marin B, Piasini E, Silver RA. LEMS: a language for expressing complex biological models in concise and hierarchical form and its use in underpinning NeuroML 2. Front Neurosci 2014;8:79. 10.3389/fninf.2014.00079 PMC4174883 25309419

[14] Gleeson P, Crook S, Cannon RC, Hines ML, Billings GO, Farinella M, et al. NeuroML: a language for describing data driven models of neurons and networks with a high degree of biological detail. PLoS Comput Biol 2010;6:e1000815. 10.1371/journal.pcbi.1000815 PMC2887454 20585541

[15] Le Novère N, Hucka M, Mi H, Moodie S, Schreiber F, Sorokin A, et al. The systems biology graphical notation. Nat Biotechnol 2009;27:735–41. 10.1038/nbt.1558 19668183

[16] Rougny A, Toure V, Moodie S, et al. Systems biology graphical notation: process description language level 1 version 2. J Integr Bioinform 2019;16:20190022. 10.1515/jib-2019-0022 PMC6798820 31199769

[17] Sorokin AA, Le Novère N, Luna A, Czauderna T, Demir E, Haw R, et al. Systems biology graphical notation: entity relationship language level 1 version 2. J Integr Bioinform 2015;12:264. 10.2390/biecoll-jib-2015-264 26528562

[18] Mi H, Schreiber F, Moodie SL, Czauderna T, Demir E, Haw R, et al. Systems biology graphical notation: activity flow language level 1 version 1.2. J Integr Bioinform 2015;12:265. 10.2390/biecoll-jib-2015-265 26528563

[19] Hucka M, Finney A, Sauro HM, Bolouri H, Doyle JC, Kitano H, et al. The systems biology markup language (SBML): a medium for representation and exchange of biochemical network models. Bioinformatics 2003;19:524–31. 10.1093/bioinformatics/btg015 12611808

[20] Hucka M, Bergmann FT, Chaouiya C, Dräger A, Hoops S, Keating SM, et al. The systems biology markup language (SBML): language specification for level 3 version 2 core release 2. J Integr Bioinform 2019;16:20190021. 10.1515/jib-2019-0021 PMC6798823 31219795

[21] Hucka M, Bergmann FT, Dräger A, Hoops S, Keating SM, Le Novère N, et al. The systems biology markup language (SBML): language specification for level 3 version 2 core. J Integr Bioinform 2018;15:20170081. 10.1515/jib-2017-0081 PMC6167032 29522418

[22] Olivier BG, Bergmann FT. SBML level 3 package: flux balance constraints, version 2. J Integr Bioinform 2018;15:20170082. 10.1515/jib-2017-0082 PMC6167036 29522419

[23] Smith LP, Hucka M, Hoops S, Finney A, Ginkel M, Myers CJ, et al. SBML level 3 package: hierarchical model composition, version 1 release 3. J Integr Bioinform 2015;12:268. 10.2390/biecoll-jib-2015-268 PMC5451323 26528566

[24] Zhang F, Meier-Schellersheim M. SBML level 3 package: multistate, multicomponent and multicompartment species, version 1, release 1. J Integr Bioinform 2018;15:20170077. 10.1515/jib-2017-0077 PMC6167033 29676994

[25] Bergmann FT, Keating SM, Gauges R, Sahle S, Wengler K. SBML level 3 package: render, version 1, release 1. J Integr Bioinform 2018;15:20170078. 10.1515/jib-2017-0078 PMC6167038 29605822

[26] Chaouiya C, Keating SM, Bérenguier D, Naldi A, Thieffry D, van Iersel MP, et al. The systems biology markup language (SBML) level 3 package: qualitative models, version 1, release 1. J Integr Bioinform 2015;12:270. 10.2390/biecoll-jib-2015-270 26528568

[27] Gauges R, Rost U, Sahle S, Wengler K, Bergmann FT. The systems biology markup language (SBML) level 3 package: layout, version 1 core. J Integr Bioinform 2015;12:267. 10.2390/biecoll-jib-2015-267 26528565

[28] Hucka M, Smith LP. The systems biology markup language (SBML) level 3 package: groups, version 1 release 1. J Integr Bioinform 2016;13:290. 10.2390/biecoll-jib-2016-290 PMC5451322 28187406

[29] Galdzicki M, Clancy KP, Oberortner E, Pocock M, Quinn JY, Rodriguez CA, et al. The synthetic biology open language (SBOL) provides a community standard for communicating designs in synthetic biology. Nat Biotechnol 2014;32:545–50. 10.1038/nbt.2891 24911500

[30] Madsen C, Goni-Moreno A, Umesh P, Palchick Z, Roehner N, Atallah C, et al. Synthetic biology open language (SBOL) version 2.3. J Integr Bioinform 2019;16:20190025. 10.1515/jib-2019-0025 PMC6798821 31199770

[31] Madsen C, Goni-Moreno A, Palchick Z, Umesh P, Roehner N, Bartley B, et al. Synthetic biology open language visual (SBOL visual) version 2.1. J Integr Bioinform 2019;16:20180101. 10.1515/jib-2018-0101 PMC6798824 31199768

[32] Waltemath D, Adams R, Bergmann FT, Hucka M, Kolpakov F, Miller AK, et al. Reproducible computational biology experiments with SED-ML – the simulation experiment description markup language. BMC Systems Biology 2011;5:198. 10.1186/1752-0509-5-198 PMC3292844 22172142

[33] Bergmann F, Cooper J, König M, Moraru I, Nickerson D, Novère NL, et al. Simulation experiment description markup language (SED-ML) level 1 version 3 (L1V3). J Integr Bioinform 2018;15:20170086. 10.1515/jib-2017-0086 PMC6167040 29550789

[34] Bergmann FT, Adams R, Moodie S, Cooper J, Glont M, Golebiewski M, et al. COMBINE archive and OMEX format: one file to share all information to reproduce a modeling project. BMC Bioinformatics 2014;15:369. 10.1186/s12859-014-0369-z PMC4272562 25494900

[35] Bergmann FT, Rodriguez N, Le Novère N. COMBINE archive specification version 1. J Integr Bioinform 2015;12:261. 10.2390/biecoll-jib-2015-261 26528559

[36] Juty N, Le Novère N, Laibe C. Identifiers. org and miriam registry: community resources to provide persistent identification. Nucleic Acids Res 2012;40:D580–6. 10.1093/nar/gkr1097 PMC3245029 22140103

[37] Courtot M, Juty N, Knüpfer C, Waltemath D, Zhukova A, Drager A, et al. Controlled vocabularies and semantics in systems biology. Molecular Systems Biology 2011;7:543. 10.1038/msb.2011.77 PMC3261705 22027554

[38] BioModels.net. 2017. http://co.mbine.org/standards/qualifiers. Accessed on 24/06/2019.

